# Body composition, muscle function, and physical performance in fibrotic interstitial lung disease: a prospective cohort study

**DOI:** 10.1186/s12931-019-1019-9

**Published:** 2019-03-12

**Authors:** Sabina A. Guler, Seo Am Hur, Scott A. Lear, Pat G. Camp, Christopher J. Ryerson

**Affiliations:** 1 Department of Medicine, University of British Columbia, Vancouver, Canada; 2 Centre for Heart Lung Innovation, University of British Columbia, Vancouver, Canada; 3 Department of Pulmonary Medicine, University Hospital and University of Bern, Bern, Switzerland; 4 Faculty of Health Sciences, Simon Fraser University, Burnaby, Canada; 5 Department of Physical Therapy, University of British Columbia, Vancouver, Canada; 6 St. Paul’s Hospital, 1081 Burrard St, Ward 8B, Vancouver, BC V6Z 1Y6 Canada

**Keywords:** Body composition, Exercise, physical, Lung diseases, Interstitial, Muscle strength, Respiratory function tests

## Abstract

**Background:**

Patients with fibrotic interstitial lung disease (ILD) are frequently physically inactive and many ILD subtypes are characterized by risk factors for myopathy; however, the importance of body composition, muscle strength, and physical performance in this population is largely unknown.

**Methods:**

Patients were prospectively recruited from a specialized ILD clinic, baseline characteristics were collected from the clinical record, pulmonary function tests were performed per established protocols, and dyspnea was measured using the University of California San Diego Shortness of Breath Questionnaire. Dual-energy X-ray absorptiometry (DXA) was used to assess body composition; handgrip strength to determine muscle strength, and 4-m gait speed to measure physical performance.

**Results:**

One hundred and fifteen patients with fibrotic ILD including 40 patients with idiopathic pulmonary fibrosis were recruited. The mean age was 69+/− 10 years in men (62% of the cohort), and 66+/− 9 years in women, with mild and moderate reduction in FVC and DLCO, respectively, for both sexes. ILD severity (measured by FVC %-predicted, DLCO %-predicted, or the Composite Physiologic Index in separate models) significantly predicted muscle mass and percent body fat including with adjustment for age, sex, and weight. ILD severity was associated with grip strength and gait speed independent from body composition.

**Conclusions:**

ILD severity has an important impact on body composition, particularly in men. Future studies are needed to confirm and further explore the possibility of additional pathways through which ILD directly impacts limb muscle function and physical performance.

## Introduction

Fibrotic interstitial lung diseases (ILDs) are progressive disorders that have high morbidity and early mortality [1, 2]. The morbidity of fibrotic ILD is primarily determined by disabling dyspnea and cough; however, extrapulmonary symptoms are also common (e.g., depression, anxiety, fatigue) [3]. Together, these pulmonary and extrapulmonary manifestations reduce physical activity [4, 5], which in turn leads to limb muscle disuse and deconditioning [6, 7]. The importance of limb muscle dysfunction on quality of life, adverse health outcomes, and mortality is well recognized in the general aging population [8], and in patients with chronic obstructive pulmonary disease (COPD) [9], but there are limited data from patients with ILD.

Beyond the impact of muscle disuse and deconditioning, there are additional potential biological links between ILD and limb muscle dysfunction. For example, many ILD subtypes are characterized by systemic inflammation, oxidative stress, hypoxemia, impaired energy balance, malnutrition, and accelerated biological aging [10–12]. These are additional potential risk factors for myopathy and limb muscle dysfunction that have also not been adequately studied in fibrotic ILD [13–15]. The overarching objective of this study was therefore to explore the importance of body composition, muscle strength, and physical performance in patients with fibrotic ILD. We hypothesized that ILD severity impacts body composition independent of baseline demographics, and that ILD severity has a direct effect on limb muscle function that is independent of muscle mass and body fat.

## Methods

### Study population

Consecutive adult patients with idiopathic pulmonary fibrosis (IPF), idiopathic nonspecific interstitial pneumonia (NSIP), chronic hypersensitivity pneumonitis (HP), and unclassifiable ILD were prospectively invited to participate from a specialized ILD clinic between January 2016 and December 2017. Diagnoses were made using established criteria where available or based on a multidisciplinary discussion for ILDs without diagnostic criteria [16, 17]. Patients with ILD secondary to a systemic disease (e.g., connective tissue disease [CTD], sarcoidosis) and patients with significant, active and/or uncontrolled cardiovascular, musculoskeletal, metabolic, malignant, or neurological disease that limited function or independence were excluded.

### Measurement of body composition, muscle function, and physical performance

Dual-energy X-ray absorptiometry (DXA) was used to assess body composition. This standard technique allows precise, accurate, and reproducible differentiation between body fat mass, lean mass, and bone mineral content, with the possibility of obtaining regional measures (e.g., upper or lower limb muscle mass) [18]. All DXA scans were performed with a Hologic Discovery QDR 4500 (Hologic Inc., Bedford, MA). As recommended by the European Working Group on Sarcopenia in Older People [19], skeletal muscle index (SMI) was calculated as the sum of the muscle mass of the upper and lower limbs adjusted for height (m^2^). Percent body fat was calculated as total fat mass divided by total body mass.

Limb muscle function was assessed by handgrip strength [19], measured to the nearest kilogram using a Baseline® HiRes™ hydraulic hand dynamometer. Patients were seated with elbows flexed to 90 degrees and forearms in neutral position, thumb facing upwards. Patients were instructed to squeeze the dynamometer as hard as possible alternating three times for each hand, with the best measurement used for all analyses. Age-matched reference values are available for the Canadian population [20].

Physical performance was assessed with 4-m gait speed (4MGS) [19], in which patients were instructed to walk at their usual pace along a 4-m course as when walking down a street. Canes and other walking aids were allowed. The walk test was performed three times with the fastest time used for all analyses. 4MGS has recently been demonstrated as reliable, valid, and responsive to change in patients with IPF [21].

### Additional measurements

Demographics, baseline characteristics, and data on prednisone use were collected from the clinical record, with all measurements performed within 3 months of recruitment. Pulmonary function tests were completed using established protocols [22, 23]. Disease severity was assessed using pulmonary physiology, including forced vital capacity (FVC), diffusion capacity of the lung for carbon monoxide (DLCO), and the Composite Physiologic Index (CPI), which was developed to predict radiological severity of fibrosis by aggregating FVC, forced expiratory volume in 1 s, and DLCO [24]. Dyspnoea was measured using the University of California San Diego Shortness of Breath Questionnaire (UCSD SOBQ), for which patients rate their dyspnea severity during 21 common activities and indicate how much dyspnea limits 3 aspects of their daily life, with each question measured on a 0–5 scale (total range 0–120) [25].

### Statistical analysis

Data are reported as mean (standard deviation) or median (interquartile range). Variables were examined for normality with histograms. Transformation of variables was not performed given the approximately normal distribution of all variables included in multivariable linear models. Between-group differences were analysed by chi^2^-test/Fisher’s exact test for categorical variables and by two-sample t-test/Wilcoxon rank-sum test for continuous variables. Unadjusted associations between continuous variables were determined by Pearson’s/Spearman’s correlation test. Multivariable linear regression models were fitted to examine the independent association of ILD severity with body composition, limb muscle function, and physical performance. The coefficient of determination (R^2^) was used to assess the percent of variance in body composition, limb muscle function, and physical performance that was explained by the linear model. Model fit was compared by the Akaike Information Criterion (AIC). A two-sided *p* < 0.05 was used to indicate statistical significance for all comparisons. Data were analyzed using R version 3.5.1 (R Foundation for Statistical Computing, Vienna, Austria) [26].

## Results

### Patient characteristics

Of the 164 consecutive patients approached, 49 declined participation and 71 men and 44 women consented to participate, with a final study cohort of 115 patients. The most frequent diagnosis was IPF (34 men and 6 women), followed by unclassifiable ILD (21 men and 16 women) and chronic HP (10 men and 16 women), representing 35, 32, and 23% of the cohort, respectively, after excluding patients with CTD-ILD and other multisystem disorders. The remaining 6 men and 6 women had cryptogenic organizing pneumonia (5), drug-induced ILD (3), vasculitis (2), idiopathic NSIP (1), and idiopathic lymphocytic interstitial pneumonia (1). On average, women were younger and had smoked less than men, with both sexes having mild reduction in FVC and moderately reduced DLCO on average (Table 1). Patients with IPF were older and had more severely impaired DLCO compared to patients with non-IPF ILD (mean [SD] age 70 [7.7] versus 66 [10] years and mean [SD] DLCO 46 [13] versus 53 [17] %-predicted, respectively). IPF and non-IPF patients had no differences in their body composition, limb muscle function, and physical performance; all ILDs were therefore pooled for all analyses.

**Table 1:** Baseline characteristics

	Men, *n* = 71	Women, *n* = 44
Demographics		
Age, years	69 (10)	66 (9)
Height, cm	169 (9)	170 (8)
Weight, kg	79 (15)	82 (16)
Body mass index, kg/m^2^	28 (4)	28 (6)
Ever smoker	48 (68%)	25 (57%)
Smoked pack-years	23 (14–37)	10 (4–17)
ILD severity		
FVC, %-predicted	77 (17)	72 (22)
DLCO, %-predicted	52 (16)	49 (17)
Dyspnea (UCSD SOBQ)	34 (22–54)	41 (23–58)
Composite Physiologic Index	45 (36–54)	48 (36–58)
Prednisone use		
Current	7 (10%)	9 (21%)
Ever	12 (17%)	17 (39%)
Dose > 10 mg daily	3 (4%)	1 (2%)

Data shown are mean (standard deviation), median (interquartile range), or number (percent)

*Abbreviations*: *DLCO* diffusion capacity of the lung for carbon monoxide, *FVC* forced vital capacity, *ILD* interstitial lung disease, *UCSD SOBQ* University of California San Diego Shortness of Breath Questionnaire

### Body composition, limb muscle function, and physical performance

Men had higher SMI (7.9 kg/m^2^ versus 6.2 kg/m^2^), higher muscle mass (total, upper limb, lower limb, and trunk), higher bone mineral content, and stronger grip compared to women (*p* < 0.001 for all comparisons). Women had higher absolute and relative body fat than men (30 kg/40% versus 25 kg/29%; *p* ≤ 0.006 for both comparisons). Gait speed did not differ significantly between men and women (1.33 m/s versus 1.25 m/s; Table 2).

**Table 2:** Body composition, limb muscle function and physical performance

	Men, *n* = 71	Women, *n* = 44	*p*-value^*^
	Mean (SD)	Mean (SD)	
Body composition			
SMI, kg/m^2^	7.9 (0.9)	6.2 (1.0)	< 0.0001
ALM, kg	24.0 (3.7)	16.2 (2.6)	< 0.0001
Upper limb, kg	6.2 (1.2)	3.6 (6.5)	< 0.0001
Lower limb, kg	17.8 (2.7)	12.6 (2.2)	< 0.0001
Trunk, kg	32.6 (5.0)	25.2 (4.1)	< 0.0001
Body fat, kg	25.1 (7.8)	29.7 (9.1)	0.006
Body fat, %	29.1 (5.2)	39.8 (6.5)	< 0.0001
Total BMC, kg	2.8 (0.6)	2.1 (0.4)	< 0.0001
Limb muscle function			
Dominant hand, kg	40.2 (9.6)	25.6 (5.7)	< 0.0001
Non-dominant hand, kg	37.5 (9.3)	24.2 (6.4)	< 0.0001
Physical performance			
4-m gait speed, m/s	1.33 (0.3)	1.25 (0.3)	0.16

*Abbreviations*: *ALM* appendicular lean mass, *BMC* bone mineral content, *SMI* skeletal muscle index

^*^Two sample t-test

**Fig. 1:**
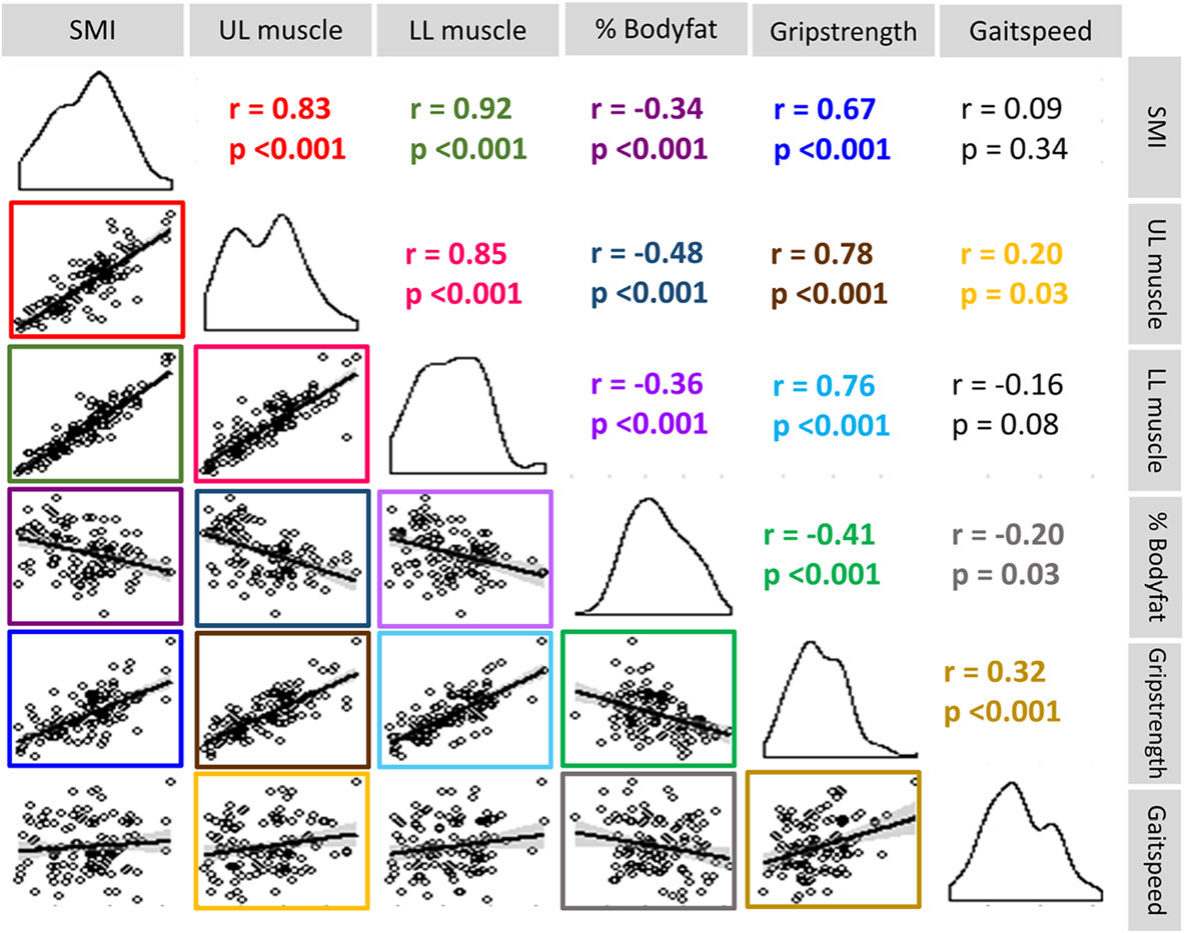
Pairwise correlation of body composition, limb muscle function, and physical performance. Abbreviations: SMI, skeletal muscle index (kg/m^2^), UL muscle, upper limb muscle mass (kg); LL muscle, lower limb muscle mass (kg); % Bodyfat, percent of fat mass/total body mass (%), Grip strength, handgrip strength (kg); Gait speed, 4-m gait speed (m/s); r, Pearson’s correlation coefficient

Figure 1 shows the correlation among measures of body composition, limb muscle function, and physical performance in men and women. SMI was correlated with age (*r* = − 0.40, *p* < 0.001) and weight (*r* = 0.69, *p* < 0.001) in men and with weight (*r* = 0.71, *p* < 0.001) in women. Percent body fat correlated with age (*r* = 0.24, *p* = 0.04) and weight (*r* = 0.63, *p* < 0.001) in men and with weight (*r* = 0.63, *p* < 0.001) in women. Grip strength correlated with age (*r* = − 0.39, *p* < 0.001) and weight (*r* = 0.46, *p* < 0.001) in men and with age (*r* = − 0.30, *p* = 0.047) and weight (*r* = 0.33, *p* = 0.03) in women. Gait speed was not associated with any baseline demographic or anthropometric measurement in men or women. Smoking history (smoked pack-years) and current or previous use of prednisone were not associated with SMI, percent body fat, grip strength, or gait speed, and these variables were therefore not considered in multivariable models.

### Association of ILD severity with body composition

**Fig. 2:**
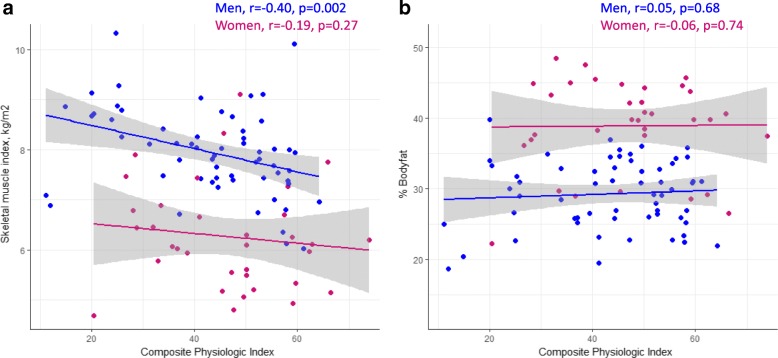
Correlation of ILD severity with body composition (**a**, skeletal muscle mass; **b**, percent body fat) in men and women with fibrotic ILD. Abbreviation: r, Pearson’s correlation coefficient

Several measures of ILD severity were associated with body composition and particularly muscle mass. On unadjusted analysis, SMI was correlated with DLCO %-predicted and CPI in men but was not associated with any measure of ILD severity in women (Table 3**,** Fig. 2a). Greater ILD severity remained a significant predictor of lower SMI in the full cohort with adjustment for age, sex, and weight, regardless of whether ILD severity was represented by FVC %-predicted, DLCO %-predicted, or the CPI. The multivariable models explained 75–76% of the variability in SMI (Table 3). On unadjusted analysis, percent body fat correlated with FVC %-predicted and dyspnea in men and with FVC %-predicted in women (Table 3**,** Fig. 2b). Lower FVC %-predicted was the only ILD severity measure that was independently associated with higher percent body fat with adjustment for age, sex, and weight on adjusted analysis (Table 3).

**Table 3:** Unadjusted and adjusted correlation of ILD severity with body composition

	Unadjusted analysis				Adjusted for sex, age, weight		
	Men		Women				
	r	*p*-value	r	*p*-value	Coefficient (95% CI)	*p*-value	R^2^
SMI, kg/m^2^							
FVC, %-predicted	0.22	0.07	−0.06	0.71	0.012 (0.005 to 0.018)	0.0006	0.75
DLCO, %-predicted	0.39	0.002	0.15	0.40	0.013 (0.006 to 0.021)	0.0009	0.76
CPI	−0.40	0.002	−0.19	0.27	−0.016 (− 0.026 to − 0.007)	0.0001	0.76
Dyspnea (UCSD SOBQ)	− 0.08	0.57	0.29	0.09	−0.004 (− 0.010 to 0.002)	0.19	0.74
Body fat, %							
FVC, %-predicted	−0.37	0.002	−0.30	0.04	−0.063 (− 0.105 to − 0.020)	0.004	0.70
DLCO, %-predicted	0.03	0.79	0.12	0.51	0.001 (−0.050 to 0.052)	0.96	0.68
CPI	0.05	0.68	−0.06	0.74	0.025 (−0.036 to 0.087)	0.42	0.69
Dyspnea (UCSD SOBQ)	0.41	0.003	0.28	0.09	0.021 (−0.017 to 0.060)	0.28	0.70

Coefficients are reported per one-unit change for the predictor variables. A positive coefficient indicates an increase in the outcome variable per unit of the predictor variable (e.g., SMI increases by an average of 0.012 kg/m^2^ for every 1% increase of FVC %-predicted)

*Abbreviations*: *CI* confidence interval, *CPI* Composite Physiologic Index, *C* diffusion capacity of the lung for carbon monoxide, *FVC* forced vital capacity, *r* Pearson’s correlation coefficient, *R*^2^ coefficient of determination (variability in the outcome explained by the model), *SMI* skeletal muscle index, *UCSD SOBQ* University of California San Diego Shortness of Breath Questionnaire

### Association of ILD severity with limb muscle function and physical performance

**Fig. 3:**
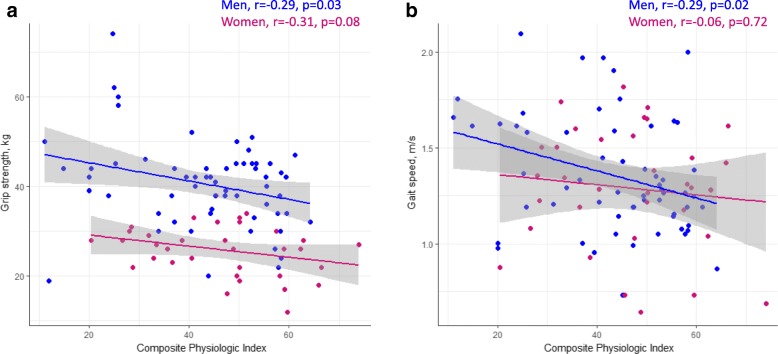
Correlation of ILD severity with limb muscle function (**a**) and physical performance (**b**) in men and women with fibrotic ILD. Abbreviation: r, Pearson’s correlation coefficient

Measures of ILD severity were associated with grip strength and gait speed independent of body composition. On unadjusted analysis, grip strength correlated with CPI in men, but was not associated with any measure of ILD severity in women (Table 4, Fig. 3a). DLCO %-predicted, CPI, and dyspnea were independent predictors of grip strength in separate models that each adjusted for age, sex, weight, and body composition. On unadjusted analysis, gait speed correlated with FVC %-predicted, DLCO %-predicted, CPI, and dyspnea in men and with dyspnea in women (Table 4**,** Fig. 3b). Gait speed was correlated with all measures of ILD severity with adjustment for baseline confounders and body composition. The models explained a greater percentage of the variability in grip strength (R^2^ 0.65–0.68) than in gait speed (R^2^ 0.07–0.16; Table 4).

**Table 4:** Unadjusted and adjusted correlation of ILD severity with physical performance

	Unadjusted analysis				Adjusted for sex, age, weight, SMI, % body fat		
	Men		Women				
	r	*p*-value	r	*p*-value	Coefficient (95% CI)	*p*-value	R^2^
Grip strength, kg							
FVC, %-predicted	0.17	0.16	− 0.13	0.41	0.050 (− 0.022 to 0.122)	0.17	0.65
DLCO, %-predicted	0.16	0.21	0.22	0.20	0.115 (0.026 to 0.204)	0.01	0.67
CPI	−0.29	0.03	− 0.31	0.08	− 0.140 (− 0.247 to − 0.034)	0.01	0.66
Dyspnea (UCSD SOBQ)	−0.08	0.58	−0.07	0.71	−0.066 (− 0.122 to − 0.010)	0.02	0.68
Gait speed, m/s							
FVC, %-predicted	0.35	0.003	0.16	0.30	0.004 (0.001 to 0.007)	0.01	0.10
DLCO, %-predicted	0.25	0.049	0.06	0.71	0.004 (0.0005 to 0.008)	0.03	0.07
CPI	−0.29	0.02	−0.06	0.72	−0.006 (− 0.011 to − 0.002)	0.009	0.08
Dyspnea (UCSD SOBQ)	− 0.28	0.04	− 0.49	0.003	− 0.005 (− 0.007 to − 0.002)	0.0003	0.16

Coefficients are reported per one-unit change for the predictor variables. A positive coefficient indicates an increase in the outcome variable per unit of the predictor variable (e.g., grip strength increases by an average of 0.115 kg for every 1% increase of DLCO %-predicted)

*Abbreviations*: *CI* confidence interval, *CPI* composite physiologic index, *DLCO* diffusion capacity of the lung for carbon monoxide, *FVC* forced vital capacity, *r* Pearson’s correlation coefficient, *R*^2^ coefficient of determination (variability in the outcome explained by the model), *SMI* skeletal muscle index, *UCSD SOBQ* University of California San Diego Shortness of Breath Questionnaire

## Discussion

In this prospective cohort study, we show that ILD severity is strongly associated with body composition in both men and women, with significantly lower muscle mass and higher fat mass in individuals with more impaired pulmonary function. We also show that ILD severity is associated with upper limb muscle dysfunction and worse physical performance independent of muscle mass or body fat percentage. The independent association of ILD severity with physical performance indicates that ILD severity has an important impact on measures of physical deconditioning in fibrotic ILD and suggests the possibility of additional pathways through which ILD directly impacts limb muscle function and physical performance. This novel finding was observed using multiple measures of ILD severity, was independent of baseline characteristics, and was not explained by the use of prednisone.

Our ILD patients had lower muscle mass and body fat percentage compared to previously published community dwelling older adults [27]. Men with fibrotic ILD also had substantially weaker grip strength compared to an age-matched healthy Canadian population, although grip strength was not lower than expected in women with fibrotic ILD [20]. These findings highlight the need for additional study of muscle mass and function in patients with fibrotic ILD. In contrast, 4MGS in our cohort was similar to the general population and markedly faster than a recently reported IPF population in the UK [21, 28]. The 4MGS is a simple, reliable, and responsive measurement for assessing global physical performance and functionality in COPD [29]; however, it was only weakly correlated with body composition in our cohort and only 10% of the variability in 4MGS was explained by ILD severity, age, sex, height, and body composition. This reflects an important contribution of unmeasured factors to 4MGS, and potentially poor construct validity of this test in our cohort.

There are two primary mechanisms by which ILD may lead to changes in muscle mass and function. First, ILD frequently leads to inactivity, muscle disuse, and deconditioning as patients attempt to avoid the uncomfortable symptom of exertional dyspnea. Second, patients with ILD frequently have risk factors for myopathy including alterations in sex and growth hormones. The impact of sex and growth hormones on muscle mass and function is well established in the general population [30, 31], and studies in COPD suggest a role of testosterone, other anabolic steroids, and myostatin in the development of age- and disease-related muscle wasting [32–34]. Similarly, patients with IPF have lower levels of the steroid hormone dehydroepiandrosterone (DHEA) [35], potentially explaining the more consistent and stronger association of ILD severity with unfavourable body composition and low physical function in men compared to women. Furthermore, DHEA has antifibrotic effects on in vitro human fibroblasts [35], indicating the need for future studies to explore the links between hormones, limb muscles, and disease progression in patients with fibrotic ILD.

Weight loss is a strong negative prognostic factor in patients with COPD [36, 37]; however, the prognostic importance of low body mass in patients with fibrotic ILD is more controversial [10, 38, 39]. Recent studies showed that erector spinae cross-sectional-muscle-area and fat free mass might be prognostically important for patients with IPF [39, 40], but neither body mass index nor body composition correlated with measures of disease severity [39]. Our novel detection of the correlation of ILD severity with body composition might be due to our larger sample size and our use of DXA to measure body composition, which is more precise at an individual patient level than bioelectrical impedance analysis that was used in the previous study [39, 41]. The prognostic significance of handgrip strength and 4MGS [21] in fibrotic ILD still needs to be determined. Studies in COPD suggest that body composition [42], muscle strength and physical performance [42–44] might be predictors of morbidity and mortality in respiratory diseases, emphasizing the potential importance of specifically targeting loss of muscle mass and limb muscle dysfunction in patients with fibrotic ILD.

Our findings have potential implications for the design of exercise training programs for patients with ILD, which can improve physical performance and reduce muscle fatigue [21, 45]. The lower muscle mass in our cohort compared to community dwelling older adults and its association with ILD severity suggest that individualized exercise programs for these patients should employ physical training strategies aimed to prevent and treat loss of muscle mass, which may further improve muscle performance. For example, sequential high intensity training of fewer muscle groups and neuromuscular stimulation may provide a sufficiently intense stimulus that can lead to muscle adaptation prior to the onset of limiting dyspnea in patients with severe ILD [46, 47]. Non-exercise strategies to prevent and treat limb muscle dysfunction have been evaluated in COPD [48], including nutritional support, oxygen therapy, and management of comorbidities. Such strategies should similarly be evaluated in the management of body composition and physical performance in patients with fibrotic ILD.

This cross-sectional study design allows only speculation about causality of the associations between ILD severity, body composition, limb muscle function, and physical performance. It is plausible that more advanced ILD leads to muscle disuse and myopathy, but common unidentified mechanisms causing myopathy and lung fibrosis can’t be excluded. Similarly, this cohort study is not able to clarify the biological mechanisms of abnormal body composition and physical performance in patients with fibrotic ILD. Finally, instead of having a direct control group, we compared body composition and physical performance measures to previously reported general populations and COPD cohorts.

## Conclusions

Maintaining independence in the growing elderly ILD population will become increasingly important in the future, particularly considering advances in ILD pharmacotherapy that might prolong survival. The development of management strategies counteracting the detrimental loss of muscle mass and function in patients with fibrotic ILD requires a deeper understanding of their physiologic basis. In this study we demonstrate a significant impact of ILD severity and dyspnea on body composition, muscle strength, and physical performance. Future studies in patients with fibrotic ILD are needed to investigate the impact of reduced muscle mass and physical function on long-term clinical outcomes, to identify potential management strategies, and to explore the underlying biological basis for these sex-dependent associations.

## Abbreviations

4MGS: 4-m gait speed
ALM: Appendicular lean mass
BMC: Bone mineral content
CI: Confidence interval
COPD: Chronic obstructive pulmonary disease
CPI: Composite Physiologic Index
CTD: Connective tissue disease
DLCO: Diffusion capacity of the lung for carbon monoxide
DXA: Dual-energy X-ray absorptiometry
FVC: Forced vital capacity
HP: Hypersensitivity pneumonitis
ILD: Interstitial lung disease
IPF: Idiopathic pulmonary fibrosis
NSIP: Nonspecific interstitial pneumonia
SMI: Skeletal muscle index
UCSD SOBQ: University of California San Diego Shortness of Breath Questionnaire
